# Masks and distancing during COVID-19: a causal framework for imputing value to public-health interventions

**DOI:** 10.1038/s41598-021-84679-8

**Published:** 2021-03-04

**Authors:** Andres Babino, Marcelo O. Magnasco

**Affiliations:** Laboratory of Integrative Neuroscience, Rockefeller University, New York, 10065 USA

**Keywords:** Data processing, Machine learning

## Abstract

During the COVID-19 pandemic, the scientific community developed predictive models to evaluate potential governmental interventions. However, the analysis of the effects these interventions had is less advanced. Here, we propose a data-driven framework to assess these effects retrospectively. We use a regularized regression to find a parsimonious model that fits the data with the least changes in the $R_t$ parameter. Then, we postulate each jump in $R_t$ as the effect of an intervention. Following the do-operator prescriptions, we simulate the counterfactual case by forcing $R_t$ to stay at the pre-jump value. We then attribute a value to the intervention from the difference between true evolution and simulated counterfactual. We show that the recommendation to use facemasks for all activities would reduce the number of cases by 200,000 ($95\%$ CI 190,000–210,000) in Connecticut, Massachusetts, and New York State. The framework presented here might be used in any case where cause and effects are sparse in time.

## Introduction

The burst of the COVID-19 pandemic forced governments around the globe to take health care interventions. One that caused significant controversies is the use of masks among the general public^1–3^. Initially, it was assumed that the primary mode of transmission of SARS-CoV-2 was through coughing or contact with surfaces. Extensive shortages of personal protective equipment for health workers led to an initial recommendation for the general population not to wear masks. While no extant studies show surgical masks reduce transmission of SARS-CoV-2 in humans, surgical masks do reduce viral shedding of other coronaviruses^4^, and transmission of SARS-CoV-2 in animal models^5^. Also, cloth masks might filter SARS-CoV-2^6^, mainly if they are worn by the infected individual by preventing droplets from being aerosolized^7^.

The Centers for Disease Control and Prevention (CDC) changed its guidelines on April 3, 2020, and recommended the widespread use of masks^8^. According to the CDC, the rationale behind its policy change was the increase in evidence that asymptomatic and presymptomatic people are infectious^9–14^ and that there are many undetected cases^15^. Similarly, The European Centre for Disease Prevention and Control (ECDC) recommends using masks^16^. Still, it states that “It is not known how much the use of masks in the community can contribute to a decrease in transmission in addition to the other countermeasures”^17^. On June 5, the World Health Organization (WHO) changed its guidelines and recommended governments encourage the general public to wear masks in specific situations like grocery stores^18^. On December 1, the WHO updated its guidance on masks and included aerosols as a means of transmission^19^. In this paper, we show evidence that the policy change regarding masks by the CDC (and local governments) decreased the number of positive cases in the states of Connecticut (CT), Massachusetts (MA), New York (NY), Rhode Island (RI), and Virginia (VA).

To assess causality, we need to evaluate both branches of an intervention^20^: one in which the intervention did happen, and one in which it did not. The gold standard to do so is the double-blind randomized control trial (RCT) paradigm. Although possible^21^, RCTs are not the norm in public health epidemiological intervention. Even if implemented, there is no such thing as a “placebo arm” for travel restrictions or a double-blind school closure. Since a placebo or double-blind trials are not possible, there is an indirect causal path between the treatment and the outcome^22,23^. For example, people in zip codes with open schools might be more careful with their hygiene because they know that they are at a higher risk than people in zip codes where the schools are closed. When the second branch of the intervention did not happen, it is called a counterfactual (“contrary to the facts”). One option to measure the direct effect of an intervention, and the one that we use in this work, is to estimate or simulate the counterfactual branch^24,25^.

Here, we present a framework to analyze data from the COVID-19 epidemic that can simulate counterfactual scenarios in which specific NPI did not occur. In this framework, we use the odds of a positive test as the dependent variable, rather than the number of positive tests^26^ or deaths^27^. We motivate a linear equation for the evolution of this dependent variable using the Susceptible-Infected-Recovered (SIR) model^28,29^. We show that we can compute the average number of people infected from one positive case on each day, a parameter known as the instantaneous reproduction number ($R_t$)^30^. Finally, we carry out a LASSO^31^ regression to fit the data, to obtain a piecewise-linear fit to the logarithm of the odds with the smallest number of breaks (see the Supplementary Fig. S4 online). This regression finds the times when interventions started, allowing us to simulate alternative scenarios where these interventions did not happen and assess their net impact.

## Results

**Figure 1:**
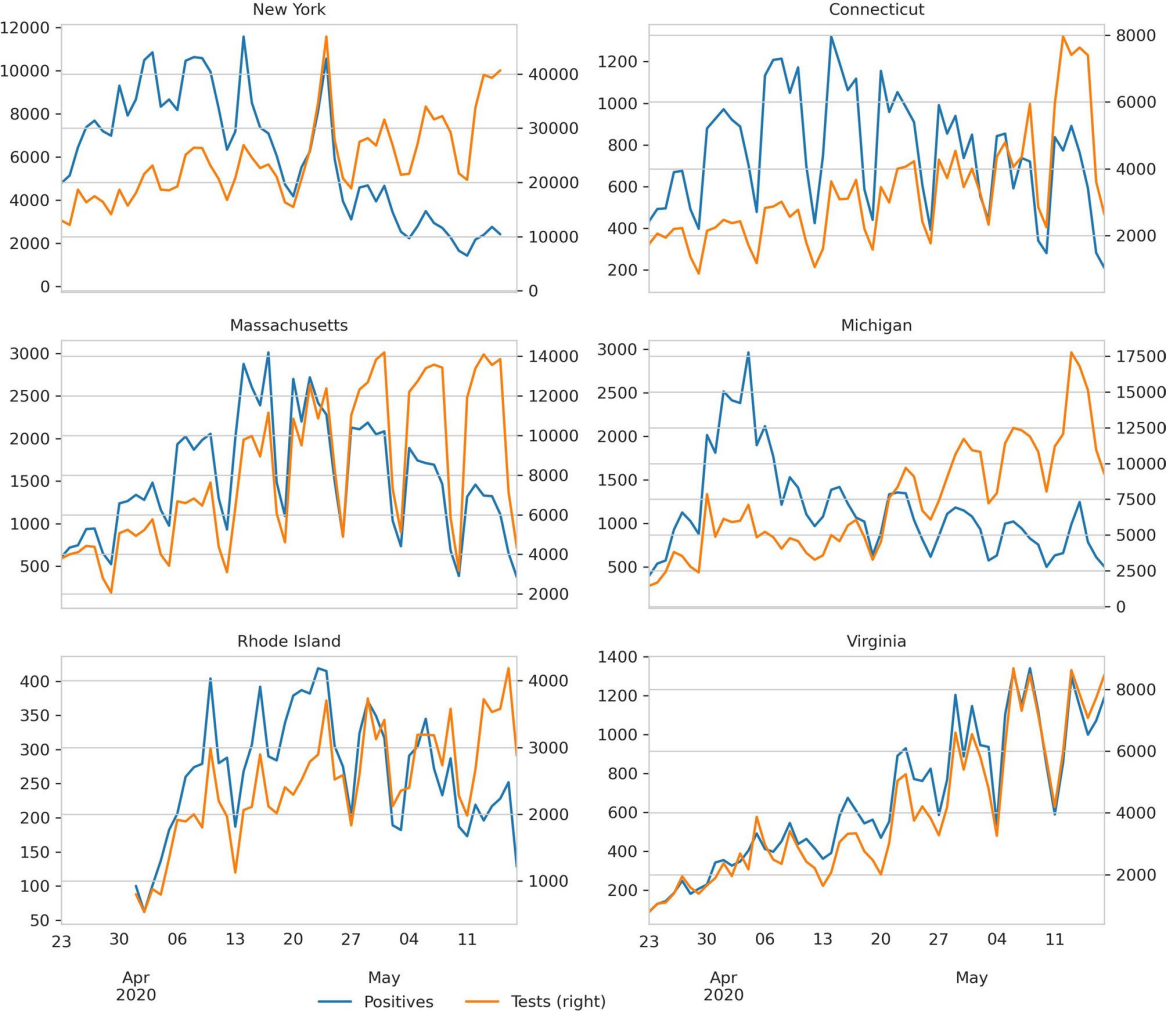
Daily number of new cases and test for each state in the dataset.

**Figure 2:**
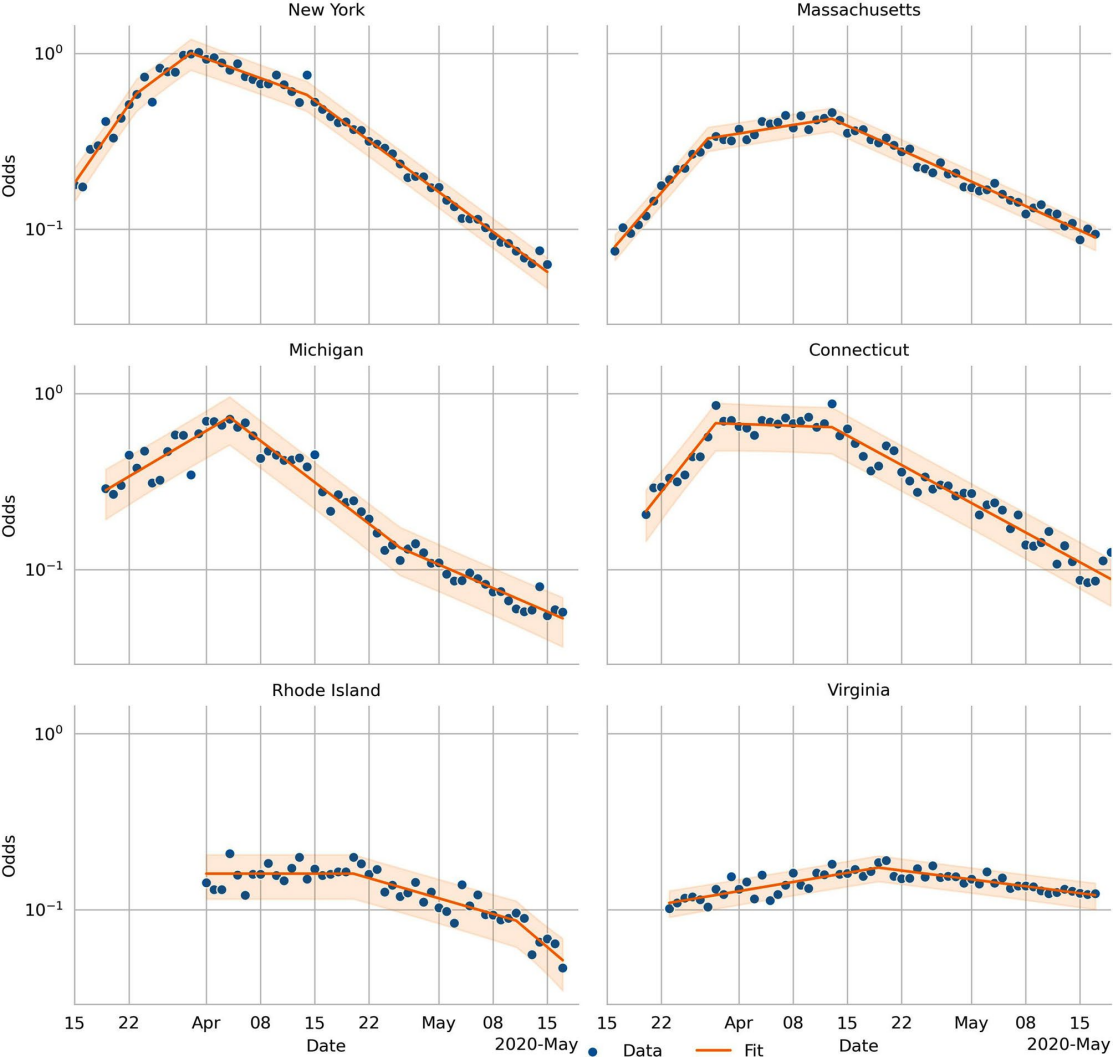
The odds of a positive test in logarithmic scale. Under the assumptions of the model, this variable should be piece-wise linear. The blue dots are the data points. The orange lines regressed model and the orange shades are is the 95% CI.

The daily number of cases and tests are highly variable (Fig. 1). To reduce this variability, we compute the *log-odds*, the logarithm of the number of positive tests over the number of negative ones. Doing this, reveals a piecewise linear pattern that we fit using the LASSO regression (Fig. 2). These regressions show three breakpoints in the *log-odds* in NY, two in CT, MA, MI, and RI, and one in VA. We should stress the fact that these breaks are not an input of the user. On the contrary, this is the result of applying the LASSO regularization. These changepoints happen after different NPIs. Once in place, the NPIs remained in effect during the period that we analyzed. The first change in CT and MA, and the first and second in NY, are due to mobility restrictions (school closure, ban mass gatherings, restriction the non-essential workforce, and stay-at-home orders). In these states, the last break happens after the CDC changed its guidelines regarding masks. In MI, RI, and VA, the stay at home orders and the CDC recommendation to wear masks happened closer in time, making it hard to disentangle their effects. However, the MI government only enforced the use of masks in closed areas (like grocery stores), and the VA government never recommended the use of masks. We assume that the lack of local orders correlates negatively with local compliance with the CDC guidelines, and that explains why we do not see the masking effect on MI and VA.

**Figure 3:**
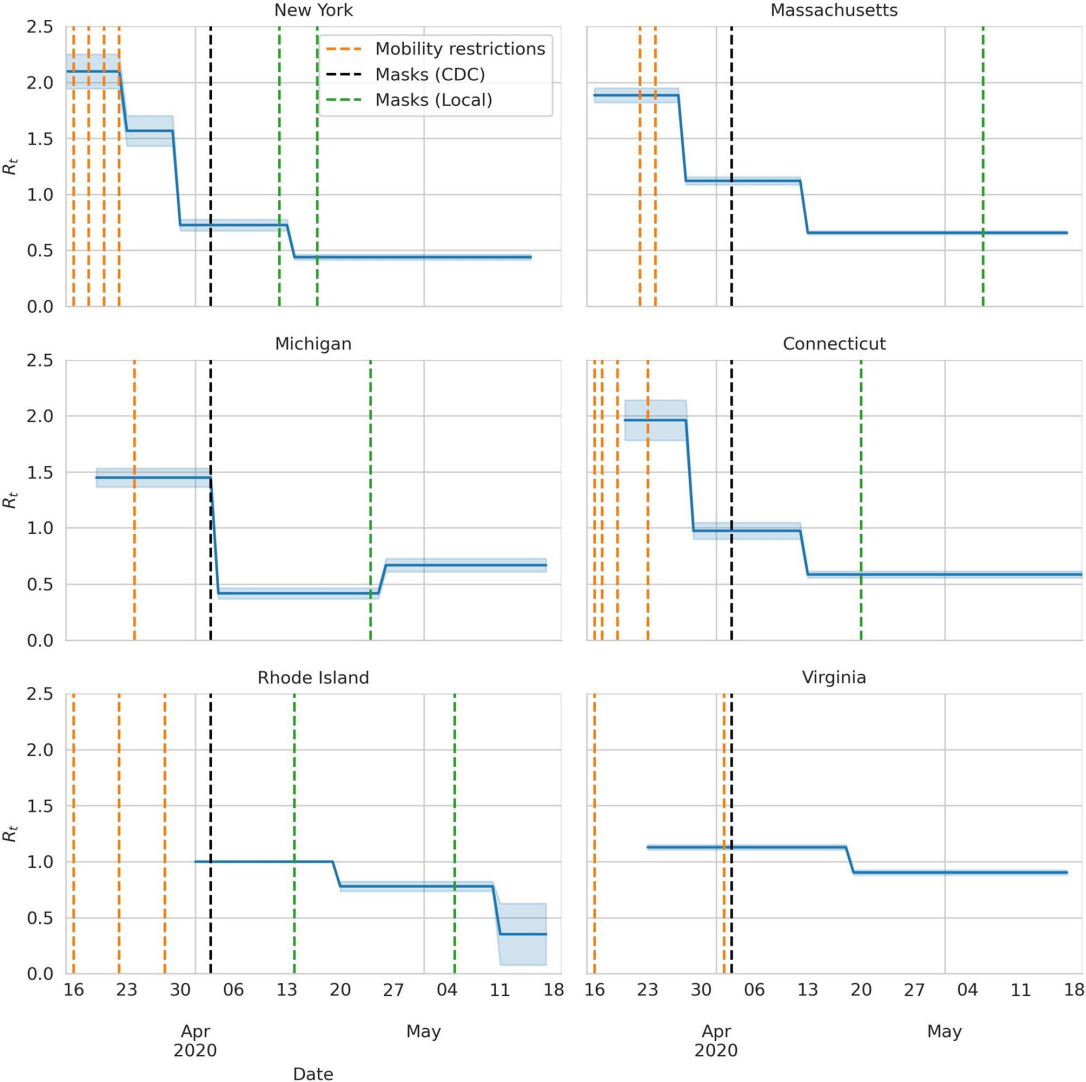
$R_t$ as a function of time. The dashed vertical lines indicate different governmental interventions. Once in place, each intervention remained in effect during the whole presented period. Orange lines indicate mobility restriction orders such as closing bars, gyms, movie theaters, schools, and banning non essential work. The black line show the moment at which the CDC updated its guidelines to recommend wearing masks. The green lines show moments at which the local states changed their guidelines regarding masks. NY and RI enforced the use of masks for some jobs first, and later on they enforced mask wearing policies among the general public. MA and CT enforced the use masks by the general public. MI only enforced the use of masks in enclosed public areas, such as grocery stores. VA never enforced the use of masks.

We use the slopes of the regression to compute the $R_t$. In Fig. 3, we show $R_t$ as a function of time, and in Tables 1 and 2 we show the values of $R_t$, the dates at which it changes, and the dates of the NPIs. The NY plot, in Fig. 3, shows $R_t$ dropping down from 2.1 to 1.6 and then to $0.72$ on March 30, 8 days after the closure of all nonessential business. Taking them together, this translates to a reduction by 65% on $R_t$ due to mobility restrictions. There is a third drop from $0.72$ to $0.44$ on April 14, 11 days after the CDC changed their guidelines and recommended to wear masks, and 2 days after NY enforced the use of masks for public employees. In CT, the stay-at-home orders reduced the value of $R_t$ by $51\%$. Moreover, after the new CDC recommendation on masks, it dropped by $40\%$. Remarkably, in MA, after the stay at home order the $R_t$ value dropped from 1.9 to 1.1, still above 1. Only after the recommendation of wearing masks it fell to 0.66, below 1. As we already mentioned, we do not see the effect of masks in MI or VA, and we attribute this to the lack of local compliance. In MI, the government only enforced the use of masks in enclosed areas, and the VA government never ruled on the use of masks. The data from RI is harder to interpret because stay-at-home orders and masks guidelines happened close in time, and data from before April are unreliable (with less than 500 tests a day). Nonetheless, there is an effect from the CDC guidelines and from local governments making masks mandatory.

**Table 1:** Values of $R_t$ for each dataset and times of $R_t$ change.

Dataset	Date	R	95% CI
New York	2020-03-15	2.10	(1.94, 2.25)
New York	2020-03-23	1.57	(1.43, 1.7)
New York	2020-03-30	0.73	(0.68, 0.78)
New York	2020-04-14	0.440	(0.416, 0.463)
Connecticut	2020-03-20	1.96	(1.78, 2.14)
Connecticut	2020-03-29	0.97	(0.9, 1.05)
Connecticut	2020-04-13	0.586	(0.557, 0.616)
Massachusetts	2020-03-15	1.89	(1.83, 1.94)
Massachusetts	2020-03-28	1.121	(1.087, 1.155)
Massachusetts	2020-04-13	0.657	(0.641, 0.674)
Michigan	2020-03-17	1.46	(1.39, 1.54)
Michigan	2020-04-04	0.415	(0.368, 0.462)
Michigan	2020-04-26	0.67	(0.61, 0.73)
Rhode Island	2020-04-01	1.07	(1.01, 1.14)
Rhode Island	2020-04-20	0.715	(0.672, 0.758)
Virginia	2020-03-23	1.128	(1.106, 1.151)
Virginia	2020-04-19	0.903	(0.881, 0.925)

**Table 2:** Events by state and date.

State	Date	Description	Type
New York	16/03/2020	Ban on large gatherings	Mobility restrictions
New York	18/03/2020	School closure	Mobility restrictions
New York	20/03/2020	Workforce up to 50%	Mobility restrictions
New York	22/03/2020	Stay at home order	Mobility restrictions
New York	03/04/2020	CDC recommends masks	Masks (CDC)
New York	12/04/2020	The state recommends masks for front line workers	Masks (Local)
New York	17/04/2020	The state recommends masks for the general public	Masks (Local)
Connecticut	12/03/2020	Ban on large gatherings	Mobility restrictions
Connecticut	16/03/2020	School closure	Mobility restrictions
Connecticut	17/03/2020	Bars closure	Mobility restrictions
Connecticut	19/03/2020	Malls closure	Mobility restrictions
Connecticut	23/03/2020	Stay at home order	Mobility restrictions
Connecticut	03/04/2020	CDC recommends masks	Masks (CDC)
Connecticut	20/04/2020	The state recommends masks for the general public	Masks (Local)
Massachusetts	22/03/2020	School closure	Mobility restrictions
Massachusetts	24/03/2020	Stay at home order	Mobility restrictions
Massachusetts	03/04/2020	CDC recommends masks	Masks (CDC)
Massachusetts	06/05/2020	The state recommends masks for the general public	Masks (Local)
Michigan	24/03/2020	Stay at home order	Mobility restrictions
Michigan	03/04/2020	CDC recommends masks	Masks (CDC)
Michigan	24/04/2020	The state recommends masks in enclosed public ...	Masks (Local)
Rhode Island	16/03/2020	Ban on large gatherings	Mobility restrictions
Rhode Island	22/03/2020	Close recreational establishments	Mobility restrictions
Rhode Island	28/03/2020	Stay at home order	Mobility restrictions
Rhode Island	03/04/2020	CDC recommends masks	Masks (CDC)
Rhode Island	14/04/2020	The state recommends masks for front line workers	Masks (Local)
Rhode Island	05/05/2020	The state recommends masks for the general public	Masks (Local)
Virginia	13/03/2020	Schools closure	Mobility restrictions
Virginia	16/03/2020	Ban on large gatherings	Mobility restrictions
Virginia	02/04/2020	Stay at home order	Mobility restrictions
Virginia	03/04/2020	CDC recommends masks	Masks (CDC)
Virginia	29/05/2020	The state recommends to wear masks indoors	Masks (Local)

**Figure 4:**
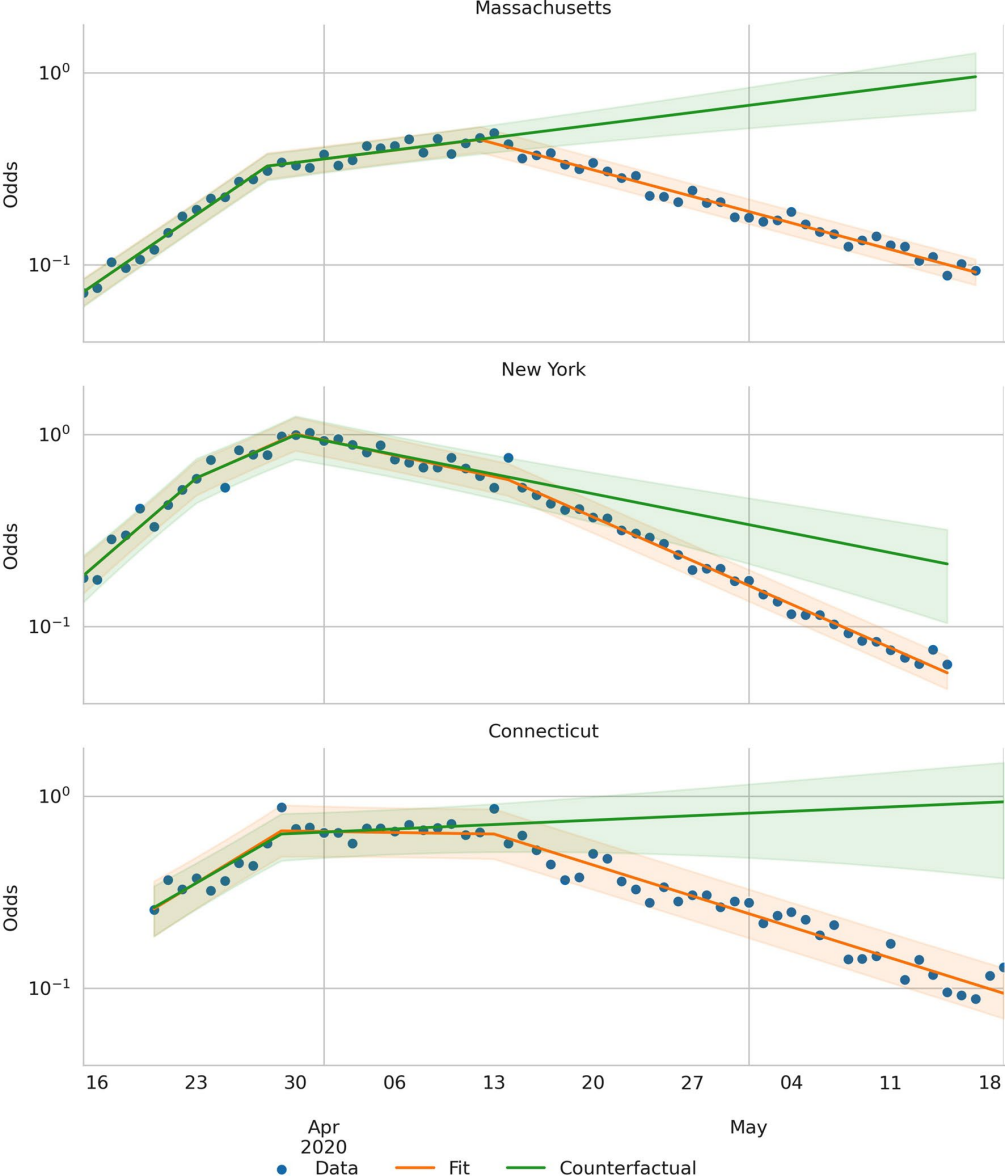
Actual and counterfactual scenarios. The blue dots are the data points. The orange lines show the regressed model, and the orange shades are the 95% CI. The green lines show the counterfactual odds of the scenario where masks were not recommended; in causal inference jargon, *do(not masks)*; and the green shaded areas are the 95% CI.

Finally, one advantage of the sparsifying framework is that we can simulate counterfactual scenarios by removing a breakpoint. Then, the regressed line would have continued at the previous slope. Take the case of the public wearing masks. From the fit shown in Fig. 4, we observe that on April 14 in NY, $R_t$ changed from $0.72$ to $0.44$. We interpret that the counterfactual to this intervention is that if the public had not used masks, $R_t$ would have stayed at $0.72$, or in causal inference jargon *do(no masks)*. Figure 4 (green line) shows that removing the intervention would have resulted in a much more drawn-out dwindling of the case curve. Now, we can use the counterfactual odds to calculate the counterfactual number of positive and compare it with the actual number of positive cases Doing this yields that, between Apr. 14 and May 15, wearing masks had the effect of decreasing the number of infections by 77,000 cases ($95\%$ CI 65,000–89,000), in NY. Similarly, the use of masks reduced the number of positive cases by 83,000 cases ($95\%$ CI 80,000–87000) in MA between Apr. 13 and May 19, and by 36,000 cases ($95\%$ CI 33,000–40000) in CT from Apr. 14 to May 17.

## Discussion

In conclusion, we found that masks reduced the spread of the virus in CT, MA, and NY. In those states, our calculations showed that the intervention reduced the $R_t$ by 40%, and we estimate that masking prevented 200,000 cases ($95\%$ CI 190,000–210,000) from the moment they were adopted until the end of the stay-at-home orders (see Table 3). These results are consistent with recently published results by Mitze et al. that found the same effect in Germany^32^. Also, we estimate that in New York City alone, masks reduced the number of cases by 29,000 ($95\%$ CI 25,000–34,000) between April 17 and May 9. This number is below the one estimated by Zhang et al. who estimated a reduction in 66,000 cases during the same period in New York City^33^. We believe the discrepancy can be accounted for by noting that Zhang et al. did not consider the increase in testing during the stay-at-home order in, leading to a higher difference after the masking order at which point the testing was more stable.

**Table 3:** Changes in $R_t$ due to each type of NPIs.

Dataset	Intervention	$R_t$% reduction	95% CI
New York	Mobility restrictions	65	(60, 70)
New York	Masks	40	(32, 47)
Connecticut	Mobility restrictions	45	(35, 55)
Connecticut	Masks	39	(31, 46)
Massachusetts	Mobility restrictions	38	(34, 42)
Massachusetts	Masks	43	(40, 46)

The framework that we presented is data-driven, and therefore it relies on only a handful of hypotheses as compared to other methods For example, the counterfactual analysis relies on one hypothesis: the *log-odds* are piecewise linear (see Eq. 6)—without the need to assume any of the hypotheses of the SIR model. Based on the goodness of fit, we are confident that this hypothesis holds for the datasets presented here (see Fig. 2 and the Supplementary Table S2 online). We also assume that the effects of the different NPIs are independent, in logarithmic scale. For example, we assume that wearing masks reduces the value of $R_t$ by the same factor, whether schools are open or not.

It should be noted that the computed $R_t$ is representative of the tested population. For example, the $R_t$ will be biased towards the one among the vulnerable populations if these are tested more often than others. Due to the structure of the social network, it might be different from the $R_t$ of the overall population. This information should be taken into account to interpret our results in a case by case basis.

Also, to put the framework to the test, we apply the method to synthetic data, and we found that it was able to find the corresponding breakpoints and slopes (see the “Supplementary Section Simulations”, Supplementary Fig. S5 and Table S4 online). Nevertheless, when there are no reliable figures on negative test results, our framework fails to fit the data (see the Supplementary Fig. S2, S3, and Table S2, online). More detailed data will be necessary to build better models in the future. Ideally, the data would be organized in a case-by-case fashion and it would contain information on the sample criterion. Fundamentally, this information should be available for negative tests results.

Overall, we found evidence that masks reduce the spread of the SARS-CoV-2 and prevent new infections. We hope that our findings will persuade local authorities and intergovernmental institutions to strongly recommend the use of masks to prevent the spread of SARS-CoV-2. We arrived at this conclusion by merging two different traditions: causal inference and regularized regression. We believe that the union of these techniques will be fruitful in other contexts where the causes and effects are sparse in time.

## Methods

### Data

We collected data from States that offer raw data on the number of tests and positive cases each day. We found that 16 States offer that information. Some of them offer an Abstraction Protocol Interface (API). Others serve a file with the information. Many states offer a visual dashboard with information on testing, but if they do not offer raw data, we did not use it. There is at least one project that collects data on testing from all the states: https://covidtracking.com/. This aggregator builds its database based on snapshots of the dashboards published by the states. The information in these snapshots is averaged three or four times a day. This process makes the accumulated number of tests and positives reliably, but not their daily change. That is why we did not use information from this aggregator, and we only use direct information from official sources. We provide links to each dataset in Table S1.

In the main text, we show results for six datasets with the highest $R^2$. To show the robustness and also limitations of the framework, we show an analysis for all the states where we found data on the daily number of cases and tests on the Supplementary Figs. S1–S3, Tables S2, and S3, online. In the main text, we limit the analysis to the time in which NYS, MA, and CT ended their stay-at-home order. To show the robustness of the framework, in the “Supplementary Information”, we show the results when we apply the method to a bigger timespan. Since, due to backlog, some states have a delay in reporting of about 1 week, we included data until the last day we find reliable data.

### The odds as the dependent variable

As can be seen in Fig. 1 the number of daily positive tests, $P\! ositive_t$, oscillates in synchrony with the number of tests. To overcome this source of noise, we propose to use the odds of a positive test:

$$\begin{aligned} O\! dds_t = \frac{P\! ositive_t}{N\! egative_t} \end{aligned}$$

where $N\! egative_t$ is the number of negative tests on day *t*.

We show the number of positives and the number of tests for each dataset in Fig. 1.

We show the evolution of the Odds in Fig. 2. The noise due to the variation in the number of tests is reduced, and a trend emerges.

### The evolution of the Odds

As shown previously^30^, under the SIR model hypotheses, the number of newly infected individuals in a given day, $k_t$, can be approximated as:

$$\begin{aligned} k_t = k_{t-1} e^{(R_{t-1}-1)\gamma } \end{aligned}$$

where $R_t$ is the instantaneous reproduction number^30^, and $\gamma ^{-1}$ is the average infectious period^29^ estimated as 7.5 days ($95\%$ CI 5.3–19) according to Li et al.^34^ (in agreement with Bi et al.^35^, but higher than reported by Du et al.^36^).

Since we do not have access to the total number of infected individuals, but only to the tested population, we have to use some statistical assumptions about this population. If we assume that the people being tested is a random sample of the population with COVID-19-like symptoms, we can state that:

1 $$\begin{aligned} P\! ositive_t = P_t(I|symptoms) P_t(symptoms) Nf_t \end{aligned}$$

where $P_t(I|symptoms)$ is the probability of a patient being positive for SARS-CoV-2 given that she is symptomatic, $P_t(symptoms)$ is the probability of having COVID-19-like symptoms, *N* is the total population, and $f_t$ is the fraction of people with symptoms that are selected to be tested (this number can be different each day, for example, if the number of tests available changes). Similarly:

2 $$\begin{aligned} N\! egative_t = P_t(not I|symptoms)P_t(symptoms)Nf_t \end{aligned}$$

where $P_t(not I|symptoms)$ is the probability of a patient being SARS-CoV-2 negative given he has COVID-19-like symptoms.

Now, if we assume that $P_t(symptoms|I)$ is constant, we can use Bayes theorem to show that:

$$\begin{aligned} P_t(I|symptoms) P_t(symptoms) \propto P_t(I) = \frac{k_t}{N} \end{aligned}$$

Then:

3 $$\begin{aligned} P_t(I|symptoms) P_t(symptoms) \propto k_t \end{aligned}$$

Finally, if we assume that $P_t(not I|symptoms)P_t(symptoms)$ is constant:

4 $$\begin{aligned} O\! dds_t = O\! dds_{t-1} e^{(R_{t-1}-1)\gamma } \end{aligned}$$

We used four sets of hypotheses. First, we use the assumptions of the SIR model. Second, we use that the tested population is a random sample from the population with COVID-19-like symptoms (Eqs. 1 and 2). This assumption does not hold, for example, if the basis for testing someone is that she was in contact with a confirmed case. If this happens, it follows that our computed $R_t$ will be biased towards this over-sampled population. For instance, it would be possible that the calculated $R_t$ would be more representative of the one among the elderly than the youth, given that the former is tested more often than the latter. Third, we assume that $P_t(not I|symptoms)P_t(symptoms)$ is constant. This hypothesis is equivalent to say that the number of people with COVID-19-like symptoms but without the SARS-CoV-2 (for example, people with the flu) is constant. Given that we compute $R_t$ in periods that span weeks, it would be enough to assume $P_t(not I|symptoms)P_t(symptoms)$ is constant during this time or that its change rate is negligible compared with the change rate in the number of symptomatic people with SARS-CoV-2. Fourth, we use that the symptoms show up instantaneously and that the tests are performed and processed on the same day (Eq. 3). This last hypothesis is not true, and it is the reason why, in our analysis, the effects of the interventions show a delay to onset between 8 and 11 days.

### Linearization

We write Eq. (4) as a linear function of the rate of change of $R_t$. Defining

5 $$\begin{aligned} b_t = e^{(R_{t}-1)\gamma } \end{aligned}$$

We can write Eq. (4) as:

6 $$\begin{aligned} O\! dds_t = b_{t-1} * O\! dds_{t-1} \end{aligned}$$

Now, instead of using $b_t$ as the parameters to estimate we decompose each $b_t$ as follows:

7 $$\begin{aligned} b_t = \prod _{i=0}^{t} a_i \end{aligned}$$

The $a_i$s represent the rate of change of the variable $b_t$ in logarithmic scale. Next, we replace the (7) in (6):

8 $$\begin{aligned} log(O\! dds_t) = \sum _{i=1}^{max(t-1, 1)} (t-i)log(a_i) + log(O\! dds_{1}) \end{aligned}$$

We can write (8) as a linear problem with the following definitions:

9 $$\begin{aligned} y\,=\, & {} X \beta + \beta _0 \end{aligned}$$

10 $$\begin{aligned} y_t\,= \,& {} log(O\! dds_t) \end{aligned}$$

11 $$\begin{aligned} X_{t,i}\,= \,& {} max(t-i, 0) \end{aligned}$$

12 $$\begin{aligned} \beta _t\,= \,& {} log(a_{t}) \end{aligned}$$

Importantly, the SIR hypotheses are only necessary to draw the connection to $R_t$ (Eq. 5). However, Eq. (8) might hold even if the SIR hypotheses do not. What would change is the interpretation of the parameters.

### LASSO regression and feature selection

Since in Eq. (9), we have as many regressors as samples, and we assume that the changes in *a* are only due to top-down interventions we use a LASSO regression to fit the data^31^. This regression minimizes the loss function:

13 $$\begin{aligned} Err = \frac{1}{n} \sum _{t=1}^n \left( y_t-\beta _0 - \sum _{i=1}^{n-1}\beta _i X_{t,i}\right) ^2 + \alpha \sum _{i=0}^{n-1}\left| \beta _i\right| . \end{aligned}$$

This approach finds a sparse set of $\beta _i$. We add two extra steps to sparsify even further this set of parameters. If there are contiguous $\beta _i \ne 0$, we set to zero all of them but the first in the chunk. Then, we fit the selected regressors using ordinary least squares, and we recursively remove the $\beta _i$ with *p*-values$*>0.01$, where *p*-values$*$ are the Bonferroni corrected *p*-values. Using the LARS algorithm^37^, we repeat these steps for different values of the hyperparameter $\alpha$, and we use the fit that minimizes the Bayesian Information Criterion^38^. We show the result of this procedure in Fig. S4.

### From fitted parameters to $R_t$

To compute the value of $R_t$ from fitter parameters, we have to use Eqs. (5, 7 and 12). From these equations, we arrive at the following equality:

14 $$\begin{aligned} R_t = \frac{\sum _{i=0}^t \beta _i}{\gamma } + 1 \end{aligned}$$

where most of the $\beta _i$ values are zero. Using this formula, we arrive at the values presented in Fig. 3 and Table 1 (main text). We show the $R_t$ values for all states with data in Fig. 3.

## Supplementary Information

Supplementary Information.

## Data Availability

All the data and codes to reproduce our analysis are publicly available at https://github.com/ababino/babino2020masks.
