# Supplementary material for: Masks and distancing during COVID-19: a causal framework for imputing value to public-health interventions
Source: Sci Rep. 2021 Mar 4;11:5183. doi: 10.1038/s41598-021-84679-8 (PMC7970858; doi:10.1038/s41598-021-84679-8)
Supplement: Supplementary file 1 — Supplementary Information. [file 41598_2021_84679_MOESM1_ESM.pdf]

Masks and distancing during COVID-19: a causal framework for imputing value to public-health interventions
Supplementary Information

Andres Babino[1,*] **and Marcelo O. Magnasco**[1]

[1]Laboratory of Integrative Neuroscience, Rockefeller University, New York, 10065, USA
[*]ababino@rockefeller.edu

Simulations

To test the framework, we show that it is able to estimate the parameters from simulated data. We use the SIR equations:

$$\frac{dS}{dt} = -\beta \frac{SI}{N} \tag{1}$$

$$\frac{dI}{dt} = \beta \frac{SI}{N} - \gamma I \tag{2}$$

$$\frac{dR}{dt} = \gamma I \tag{3}$$

where S is the number of susceptible individuals, I the number of infected individuals, R the number of recovered (or death) individuals, β is the contact rate and γ the inverse of the average infectious period. Also, $R_0 = \beta/\gamma$

In our simulation we change the value of β at times $t = 21$, $t = 41$, and $t = 61$, from a initial value of $\beta = 0.26$ to $\beta = 0.2$, and then to $\beta = 0.093$ and $\beta = 0.053$. Also we set $\gamma = 7.5^{-1}$ We chose these values because the R_t are similar to the one in the NYS dataset. After carrying out the simulation we compute the *log-odds*= $log(I/S)$ and add noise ε $N(0, 0.05)$ We show the results applying the framework to this data in Fig. S5 and the real and estimated values in Table S4. Initially, the framework estimates $R_t = 1.7$ $(1.95, 2.0)$ 95% CI. This value is below the $R_0 = 2$ of the model, which is reasonable given that the framework should estimate the mean value of R_t in a segment, which is always below R_0 (26). The second discontinuity is found at $t = 39$ and last one at $t = 59$, two days before the real change.

This analysis shows that the framework can detect the number of discontinuities correctly, and it also estimates the value of R_t accurately and the times of the breaks with no more than 2 days of error.

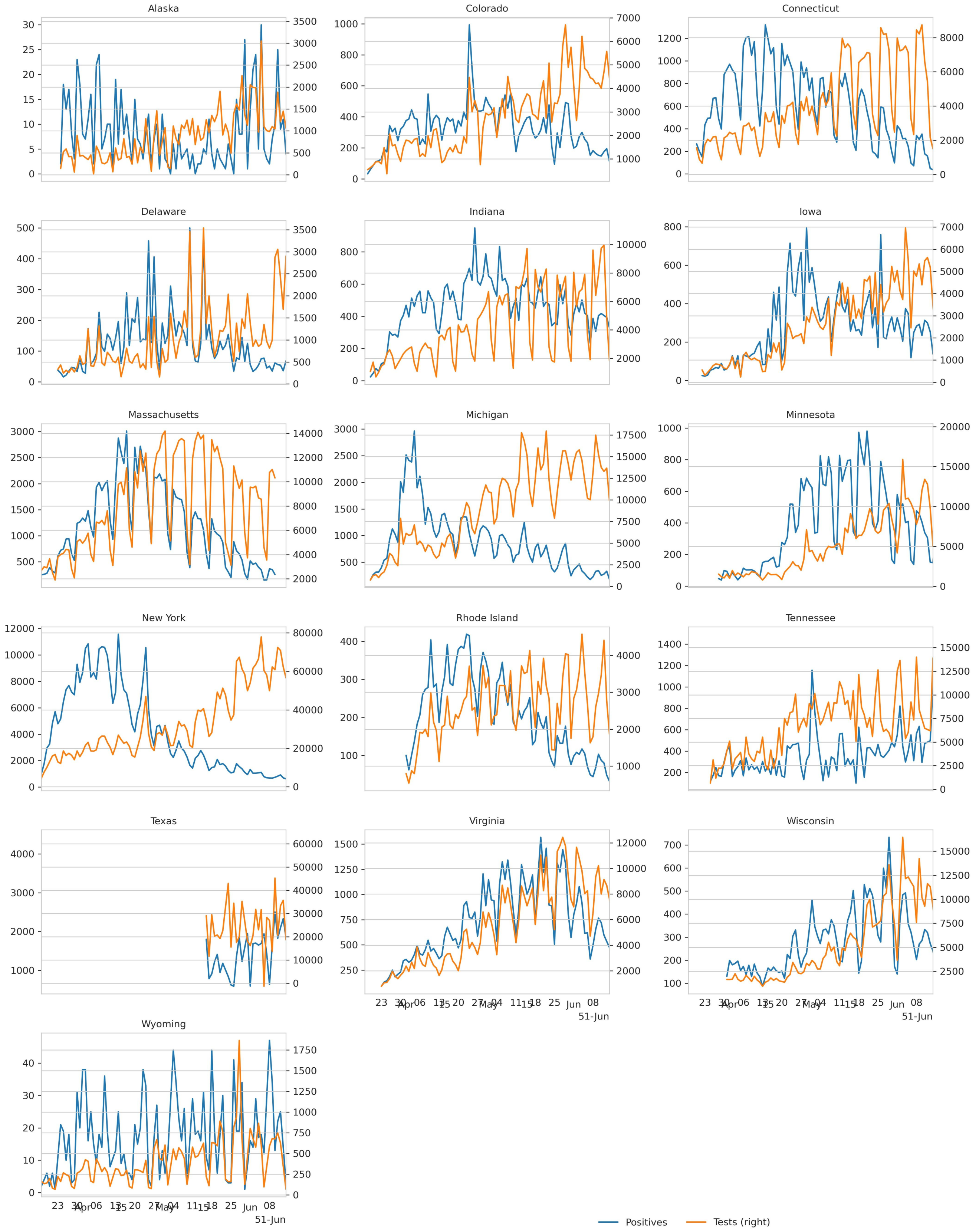

Figure S1. Daily number of new cases and tests for each state in the dataset.

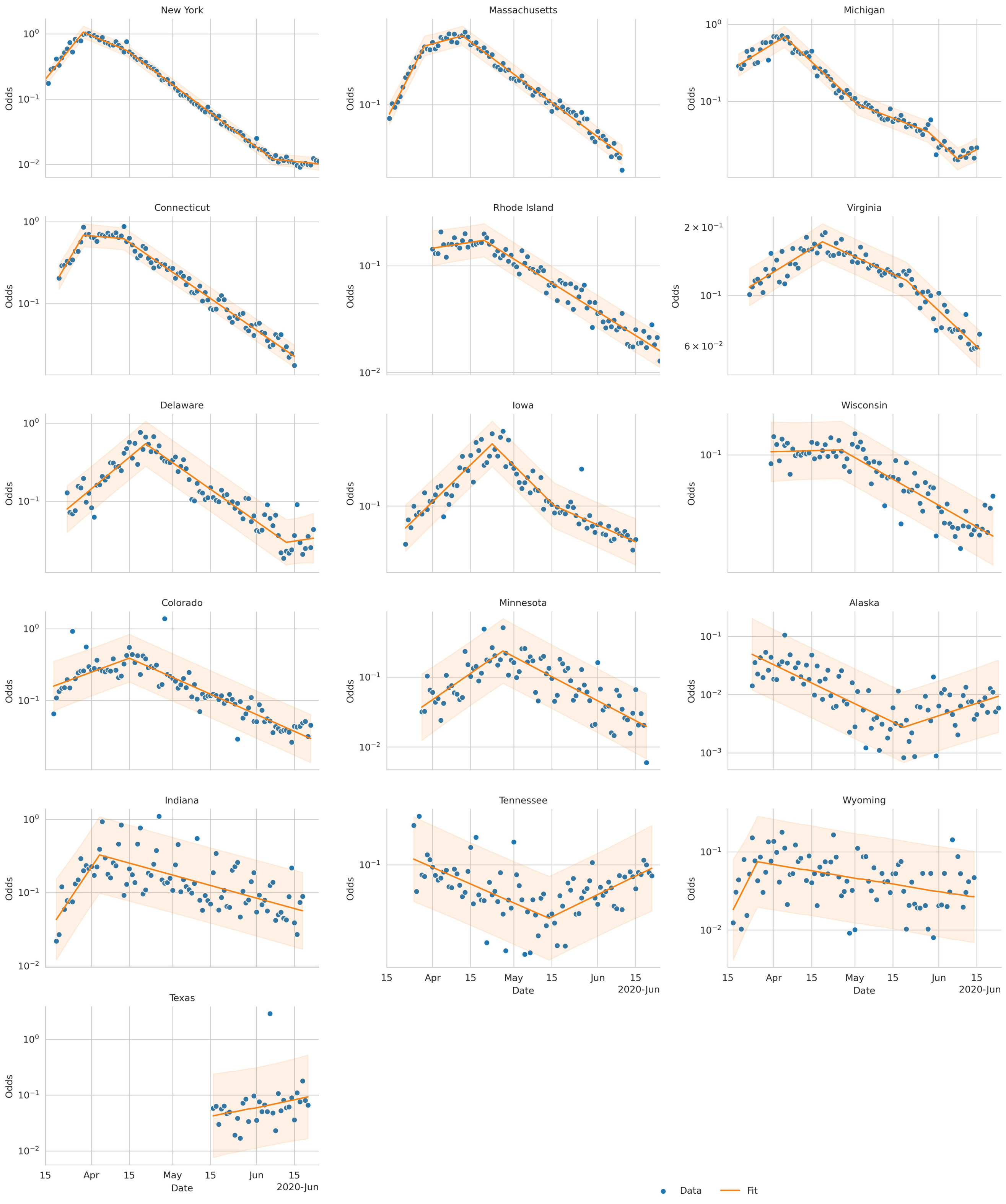

Figure S2. The odds of a positive test in logarithmic scale. Under the assumptions of the model, this variable should be piecewise linear. The blue dots are the data points. The orange line is the LASSO fit, and the orange shade is the 95% C.I.

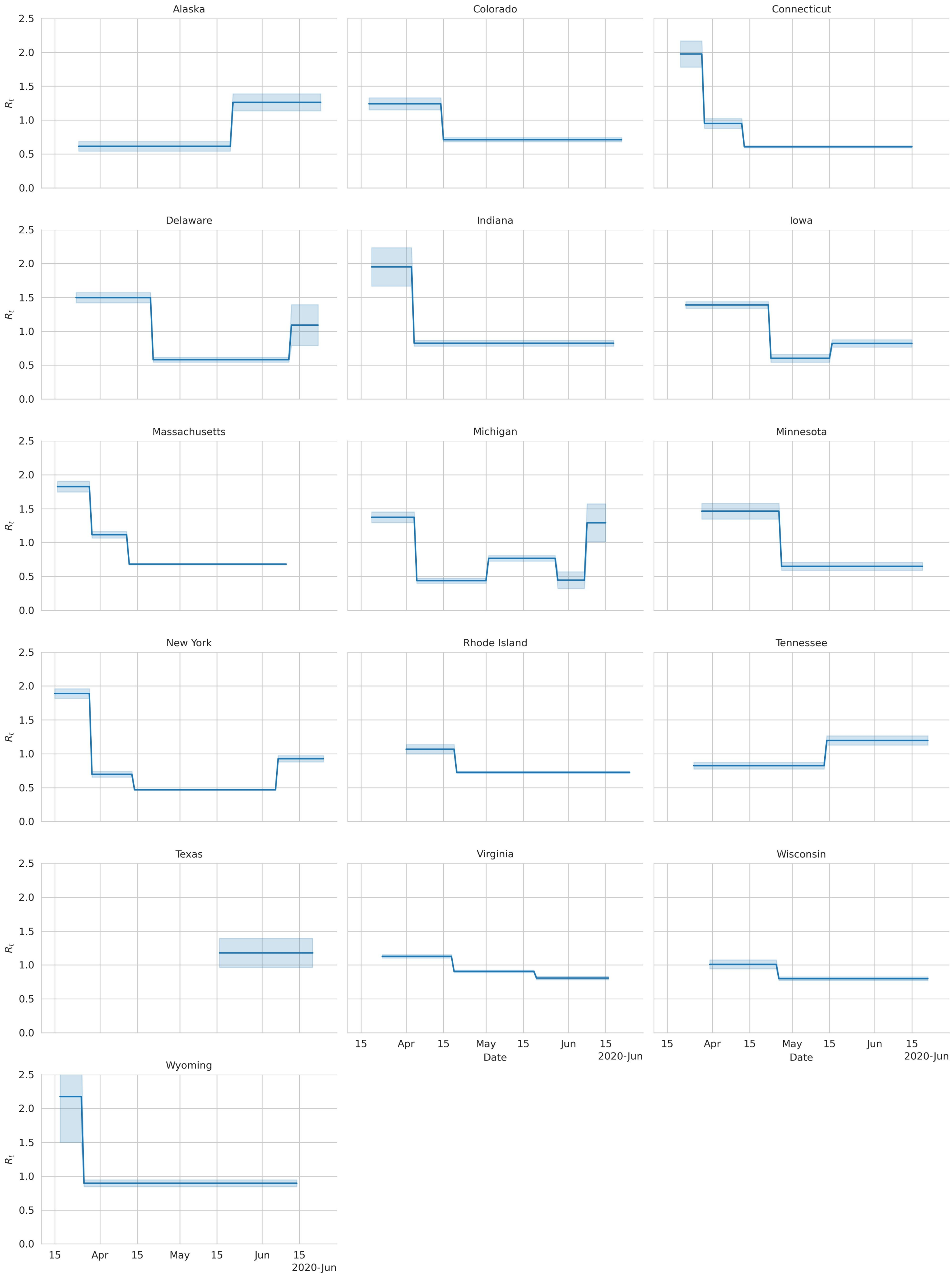

Figure S3. R_t as a function of time.

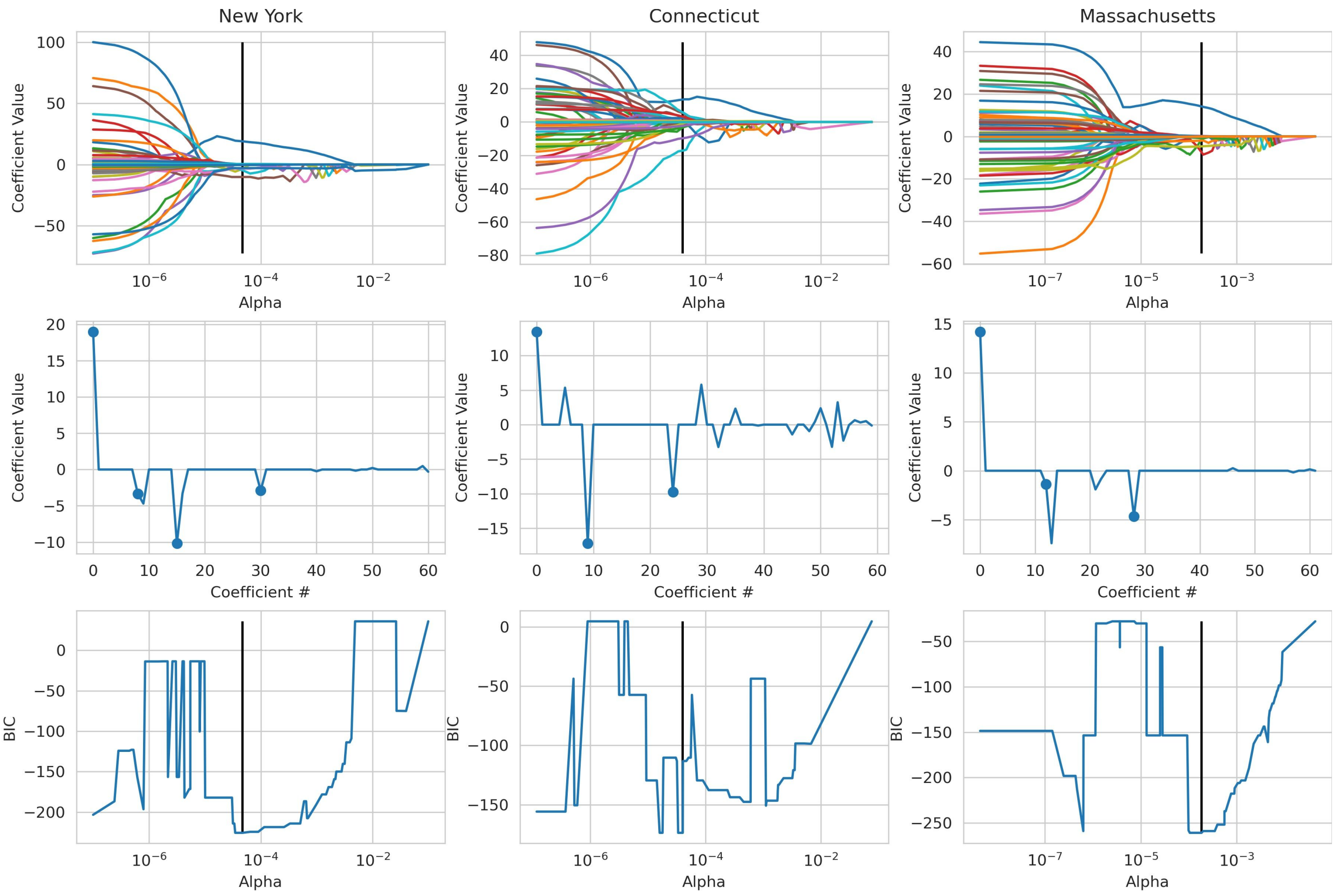

Figure S4. Steps of the framework. First row: coefficient values as a function of the parameter α. As the value of α increases, the parameters collapse to zero. The black vertical line shows the selected value of α. Second row: coefficient values at the selected α. At the final α, most of the coefficients are zero; if two of them are non zero in a row, we select the first one in the chunk. With the selected coefficients, we carry out a linear fit, and we iteratively remove the coefficients with *p-value*$^{*} < 0.01$. The dots indicate the parameters that were selected, either for being the first of a chunk or being significant. Third row: BIC as a function of α. Since we performed these steps for all the α values, we compute the BIC for each of them. Then, we pick the one with the lowest BIC value.

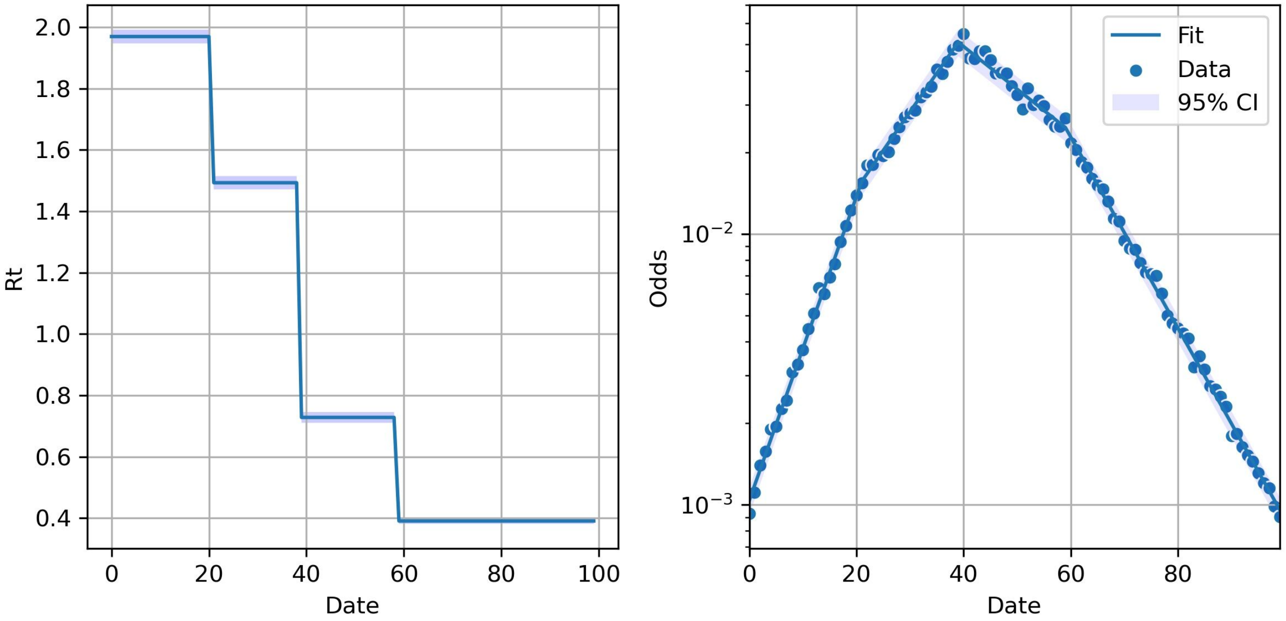

Figure S5. Simulated data using the SIR equations and the results from applying the framework. Left panel: estimated R_t as a function of time. Right panel: simulated odds and final fit.

Table S1. Information about the source of the datasets used in this work

Dataset	Link to information
Alaska	https://coronavirus-response-alaska-dhss.hub.arcgis.com/datasets/daily-test-positivity/data
Colorado	https://data-cdphe.opendata.arcgis.com/datasets/cdphe-covid19-daily-state-statistics/data
Connecticut	https://data.ct.gov/Health-and-Human-Services/COVID-19-PCR-Based-Test-Results-by-Date-of-Specime/qfkt-uahj
Delaware	https://myhealthycommunity.dhss.delaware.gov/locations/state
Indiana	https://hub.mph.in.gov/dataset/covid-19-case-trend/resource/182b6742-edac-442d-8eeb-62f96b17773e
Iowa	https://coronavirus.iowa.gov/
Massachusetts	https://www.mass.gov/info-details/covid-19-response-reporting
Michigan	https://www.michigan.gov/coronavirus/0,9753,7-406-98163_98173—,00.html
Minnesota	https://www.health.state.mn.us/diseases/coronavirus/situation.html
New York	https://health.data.ny.gov/Health/New-York-State-Statewide-COVID-19-Testing/xdss-u53e
Rhode Island	https://docs.google.com/spreadsheets/d/1n-zMS9Al94CPj_Tc3K7Adin-tN9x1RSjjx2UzJ4SV7Q/edit#gid=590763272
Tennessee	https://www.tn.gov/health/cedep/ncov/data/downloadable-datasets.html
Texas	https://www.dshs.texas.gov/coronavirus/
Virginia	https://www.vdh.virginia.gov/coronavirus/
Wisconsin	https://data.dhsgis.wi.gov/datasets/covid-19-historical-data-table/data?where=GEO%20%3D%20%27State%27
Wyoming	https://health.wyo.gov/publichealth/infectious-disease-epidemiology-unit/disease/novel-coronavirus/covid-19-testing-data/

Table S2. Goodness of fit for each dataset.

State	R^2	N	D.F.	F-value	p-value
New York	0.996	102	4	6308.081088	2.989901e-116
Massachusetts	0.984	87	3	1666.265804	5.057106e-74
Michigan	0.983	89	5	987.866820	2.324393e-72
Connecticut	0.981	88	3	1438.831575	4.534263e-72
Rhode Island	0.952	85	2	810.885200	9.517308e-55
Virginia	0.918	86	3	305.468584	2.184829e-44
Delaware	0.886	92	3	228.607283	2.038413e-41
Iowa	0.872	86	3	185.719315	1.862787e-36
Wisconsin	0.811	83	2	171.824297	1.105016e-29
Colorado	0.804	96	2	191.334575	1.095911e-33
Minnesota	0.640	84	2	71.849515	1.131865e-18
Alaska	0.589	89	2	61.705125	2.401719e-17
Indiana	0.457	92	2	37.520871	1.521898e-12
Tennessee	0.398	88	2	28.101998	4.283584e-10
Wyoming	0.203	84	2	10.312295	1.023745e-04
Texas	0.079	34	1	2.752898	1.068493e-01

Table S3. Parameter values and statistics for the selected model for each dataset

Dataset	Coef. Name	Coefficient	95% C.I.	p-value
New York	x1	0.146	$(0.126, 0.167)$	1.94e-20
New York	x2	-0.071	$(-0.106, -0.036)$	1.60e-04
New York	x3	-0.112	$(-0.135, -0.089)$	7.51e-14
New York	x4	-0.038	$(-0.047, -0.029)$	8.96e-12
New York	const	-1.70	$(-1.81, -1.59)$	4.50e-37
Connecticut	x1	0.128	$(0.104, 0.152)$	2.90e-15
Connecticut	x2	-0.132	$(-0.163, -0.101)$	9.93e-12
Connecticut	x3	-0.052	$(-0.064, -0.039)$	2.57e-11
Connecticut	const	-1.54	$(-1.70, -1.39)$	6.68e-27
Massachusetts	x1	0.118	$(0.110, 0.126)$	2.02e-38
Massachusetts	x2	-0.102	$(-0.113, -0.091)$	2.55e-26
Massachusetts	x3	-0.062	$(-0.068, -0.056)$	1.43e-28
Massachusetts	const	-2.65	$(-2.72, -2.58)$	5.36e-61
Michigan	x1	0.062	$(0.052, 0.071)$	5.51e-19
Michigan	x2	-0.140	$(-0.154, -0.126)$	2.03e-27
Michigan	x3	0.034	$(0.022, 0.047)$	1.22e-06
Michigan	const	-1.42	$(-1.54, -1.30)$	1.67e-31
Rhode Island	x1	0.010	$(0.001, 0.018)$	3.12e-02
Rhode Island	x2	-0.048	$(-0.061, -0.035)$	2.45e-09
Rhode Island	const	-1.93	$(-2.04, -1.81)$	7.02e-33
Virginia	x1	0.0171	$(0.0141, 0.0202)$	1.21e-15
Virginia	x2	-0.030	$(-0.035, -0.025)$	1.18e-15
Virginia	const	-2.22	$(-2.27, -2.16)$	1.24e-56

Table S4. Simulated and estimated values of R_t

t	β/γ	$\hat{t}$	R_t	95% *CI*
0	2	0	1.97	$(1.95, 1.99)$
21	1.5	21	1.50	$(1.47, 1.51)$
41	0.7	39	0.73	$(0.71, 0.75)$
61	0.4	59	0.39	$(0.38, 0.40)$
